# Identification and Characterization of c-di-GMP Metabolic Enzymes of *Leptospira interrogans* and c-di-GMP Fluctuations After Thermal Shift and Infection

**DOI:** 10.3389/fmicb.2018.00764

**Published:** 2018-04-20

**Authors:** Guohui Xiao, Liangliang Kong, Rongbo Che, Yusi Yi, Qinchao Zhang, Jie Yan, Xu'ai Lin

**Affiliations:** ^1^Department of Medical Microbiology and Parasitology, School of Medicine, Zhejiang University, Hangzhou, China; ^2^Zhejiang Tianke High Technology Development CO. Ltd., Hangzhou, China; ^3^Basic Medical Microbiology Division, State Key Laboratory for Diagnosis and Treatment of Infectious Diseases, School of Medicine, Zhejiang University, Hangzhou, China

**Keywords:** *Leptospira*, c-di-GMP, diguanylatecyclases, phosphodiesterases, gene expression, infection

## Abstract

Leptospirosis is a widespread zoonotic disease caused by pathogenic *Leptospira* species. The most common species, *Leptospira interrogans*, can transfer from contaminated soil or water to the human body. It is able to survive these changing environments through sensing and responding to the changes of environmental cues. Cyclic di-GMP (c-di-GMP) is a special secondary messenger in bacteria, which can respond to the environment and regulate diverse bacterial behaviors. The c-di-GMP levels in bacterial cells are regulated by diguanylatecyclases (DGC) and phosphodiesterases (PDE), which are responsible for synthesizing or hydrolyzing c-di-GMP, respectively. In this study, distribution and phylogenetics of c-di-GMP metabolic genes among 15 leptospiral species were systematically analyzed. Bioinformatics analysis revealed that leptospiral species contain a multitude of c-di-GMP metabolic genes. C-di-GMP metabolic genes in *L. interrogans* strain Lai 56601 were further analyzed and the results showed that these genes have very diverse expression patterns. Most of the putative DGCs and PDEs possess enzymatic activities, as determined by riboswitch-based dual-fluorescence reporters *in vivo* or HPLC *in vitro*. Furtherer analysis of subdomains from GGDEF-containing proteins revealed that the ability to synthesize c-di-GMP was lost when the GAF domain from LA1483 and PAS domain from LA2932 were deleted, while deletion of the REC domain from LA2528 did not affect its ability to synthesize c-di-GMP. Furthermore, high temperatures generally resulted in low c-di-GMP concentrations in *L. interrogans* and most of the c-di-GMP metabolic genes exhibited differential temperature regulation. Also, infection of murine J774A.1 cells resulted in reduced c-di-GMP levels, while no significant change of c-di-GMP metabolic genes on transcriptional levels were observed during the infection of J774A.1 cells. Taken together, these results provide a basic platform for future studies of c-di-GMP signaling pathways in *Leptospira*.

## Introduction

Leptospirosis is a globally distributed emerging zoonotic disease that is caused by spirochetes of the pathogenic *Leptospira* species (Levett, 2001; Bharti et al., 2003). Leptospires are thin, highly motile, and grown under aerobic conditions with an optimum growth range *in vitro* of 28–30°C (Li et al., 2000). Some *Leptospira* species are able to survive in different conditions, such as in soil, water and in different host cells (Chang et al., 1948; Okazaki and Ringen, 1957; Crawford et al., 1971). During an infection, the growth environment of leptospires, including temperature, oxygen levels, acidity and hypertonicity, are changed compared with free-living conditions. Leptospires might possess signal pathways to sense the different external environments and adapt to changing environmental conditions.

The secondary messenger c-di-GMP is an ubiquitous signaling molecule in bacterial species and it has been shown to regulate biofilm formation, cell cycle, motility, chemotaxis, and virulence (Römling et al., 2013). Diguanylatecyclases (DGCs) possessing a GGDEF domain are responsible for synthesizing c-di-GMP, while phosphodiesterases (PDEs) containing an EAL or HD-GYP domain hydrolyze c-di-GMP. The intact GG[D/E]EF motif of GGDEF domain proteins, the EXLXR motif of EAL domain proteins and the HD residues and HHExxDGxxGYP motif of HD-GYP domain proteins are important for conferring enzymatic activity (Wassmann et al., 2007; Rao et al., 2008; Lovering et al., 2011; Bellini et al., 2014). Most of the DGCs and PDEs tend to couple with diverse regulatory or sensory domains, including PAS, GAF, and REC, and regulate the DGC or PDE activity by sensing O_2_, NO, light or a variety of other external environmental signals (Tarutina et al., 2006; Tuckerman et al., 2009; Sawai et al., 2010). Various c-di-GMP receptors have already been identified, including PilZ domain-based proteins, GGDEF I-site-based proteins, EAL domain-based proteins and some other proteins that do not have typical c-di-GMP-binding structures (Chou and Galperin, 2016). In addition, two class of riboswitches, c-di-GMP-I and c-di-GMP-II, have been reported to interact with c-di-GMP and contribute to gene regulation (Sudarsan et al., 2008; Lee et al., 2010). Furthermore, several studies revealed that the mammalian innate immune system can directly sense c-di-GMP through specific receptors, thereby initiating an emerging research area on the role of c-di-GMP in host-microbe interaction (Burdette et al., 2011; Yin et al., 2012; Li et al., 2015).

Thus far, only one paper published by da Costa Vasconcelos and his colleagues reported a GGDEF domain protein of *L. interrogans* serovar Copenhageni possesses DGC activity *in vitro* (da Costa Vasconcelos et al., 2017). To understand and uncover the role of c-di-GMP signaling pathways in the leptospiral lifecycle, characterization of enzymes responsible for synthesis or degradation of c-di-GMP is essential.

In this study, distribution and phylogeny of c-di-GMP metabolic genes were analyzed in the genomes of 15 *Leptospira* species, including 6 pathogenic species (*L. borgpetersenii, L. santarosai, L. interrogans, L. kirschneri, L. noguchii*, and *L. weilii*), 5 intermediate pathogenic species (*L. wolffii, L. fainei, L. licerasiae, L. broomii*, and *L. inadai*) and four saprophytic species species (*L. biflexa, L. wolbachii, L. meyeri*, and *L. yanagawae*). C-di-GMP metabolic genes from *L. interrogans* were further analyzed and enzymatic activities of all putative DGCs and PDEs of *L. interrogans* were verified by *in vivo* or *in vitro* methods. Furthermore, the effect of temperature on c-di-GMP levels was analyzed and the variation of c-di-GMP levels was determined during infection of mouse J774A.1 cells.

## Materials and methods

### Bioinformatics analysis

Proteins containing GGDEF, EAL, or HD-GYP domains in *L. interrogans* strain Lai 56601 were obtained from the database available at https://www.ncbi.nlm.Nih.gov/CompleteGenomes/c-di-GMP.html (Römling et al., 2013). Protein sequences were subsequently used to screen c-di-GMP metabolic proteins in the other 14 fully sequenced *Leptospira* species available at GenBank using blastP with an E-value threshold of 0.001. Conserved domains were predicted using the Conserved Domain Database (https://www.ncbi.nlm.nih.gov/cdd/) and Pfam (http://pfam.xfam.org/). Proteins lacking GGDEF, EAL, or HD-GYP domains were excluded from further analyses. Multiple sequence alignments were performed using ClustalW. Phylogentic trees were generated using the maximum-likelihood method in PhyML version 3.1. Phylogenetic trees were annotated using EvolView (http://www.evolgenius.info/evolview/#login) (He et al., 2016).

### Strains, culture conditions, and plasmids

*Leptospira interrogans* strain Lai 56601 was obtained from the National Institute for the Control of Pharmaceutical and Biological Products, Beijing, China, and grown in Ellinghausen-McCullough-Johnson-Harris (EMJH) medium at 28°C under aerobic conditions. *Escherichia coli* strains DH5α and BL21 (DE3) were cultivated in lysogeny broth (LB) medium or LB agar plates at 37°C. Antibiotics were added to the medium when required as follows: Ampicillin (100 μg/mL), Kanamycin (50 μg/mL), Spectinomycin (100 μg/mL), Erythromycin (10 μg/mL) and Lincomycin (25 μg/mL). The pET-28a and pET-32a vectors were maintained in our lab. pET-*pleD* and riboswitch-based dual-fluorescence reporter plasmids: pRP0122-*Pbe-amcyan_Bc3-5-_turborfp* and pRP0122-*Pbe-amcyan_Bc3_turborfp* were donated by professor He from Huazhong Agricultural University, China (Zhou et al., 2016). The engineered *Bacillus subtilis* strain, which is devoid of c-di-GMP metabolic enzymes, and pXG101, which is used for homologous recombination, were donated by professor Kearns and professor Dann from Indiana University (Gao et al., 2013, 2014).

### Construction of plasmids

C-di-GMP metabolic genes were amplified from *L. interrogans* genomic DNA by PCR, cloned into the pGEM-T-easy vector and confirmed by sequencing. The correct DNA fragments were subsequently sub-cloned into pET-28a or pET-32a. Truncated LA2932 (GGDEF domain), truncated LA1483 (GGDEF domain) and truncated LA2528 (GGDEF domain) were also amplified by PCR and cloned into pET-28a. Primers used for PCR amplification are listed in Table S1.

### Expression and purification of target proteins

Target recombinant expression plasmids were transformed into *E. coli* BL21 (DE3) and cultivated on LB agar with antibiotics at 37°C. The transformed bacteria were inoculated into 10 mL LB medium with antibiotics and incubated overnight at 37°C with shaking. Cultures were diluted into 1 L fresh LB medium and incubated at 37°C with shaking until the OD_600_ reached 0.6~0.8 (about 3 h). Subsequently, 0.5 mM isopropyl β-D-thiogalactoside (IPTG) was added and cultures were incubated at 37°C for 4 h or 25°C for 12 h. Bacteria were harvested by centrifugation at 12,000 g for 10 min and frozen at −80°C. Bacterial pellets were suspended into 0.01 M PBS to a total volume of 150 mL and lysed by ultrasonication. Bacterial debris was removed by centrifugation at 12,000 g and the supernatant was diluted with 50 mL purified buffer (20 mM Tris-HCl, pH 7.9, 0.5 M NaCl, 10% Glycerol). The recombinant proteins were purified using Ni-NTA superflow resin (Biocolors, Shanghai, China) according to the manufacturers' instructions. The purified proteins were analyzed by SDS-PAGE. Concentrations of the purified proteins were determined using the BCA protein assay reagent kit (Beyotime, Shanghai, China).

### Enzyme activity analysis of DGCs and PDEs

*In vivo* assays: c-di-GMP riboswitch-based dual-fluorescence reporter plasmids pRP0122-*Pbe-amcyan_Bc3-5_turborfp* and pRP0122-*Pbe-amcyan_Bc3_turborfp*, invented by Zhou and his colleagues (Zhou et al., 2016), were used to determine activities of DGCs and PDEs. The relative fluorescence intensity (RFI), defined as the ratio between the fluorescence intensities of TurboRFP at 574 nm and AmCyan at 489 nm, shows a linear correlation with intracellular c-di-GMP levels (Zhou et al., 2016). Enzymatic activities are determined according to the RFI values. The experimental procedures were performed as published previously (Zhou et al., 2016). The recombinant plasmids, containing DGCs or PDEs encoding genes and dual-fluorescence reporter plasmid, were co-transformed into *E. coli* BL21 (DE3) to obtain reporter strains. The reporter strains were cultivated in LB medium at 37°C until an OD_600_ of 0.8 was reached. 1.0 mM IPTG was added and cultures were incubated for 20 h at 28°C. For observing color changes by bright field microscopy, cultures were concentrated 5-fold, resuspended in water and the color intensity was subsequently recorded with a digital camera. To determine the RFI, cultures were diluted to an OD_600_ of 0.1 in water. Fluorescence spectra were detected using a Spectramax M5 (Molecular Devices, Sunnyvale, CA, USA).

A modified *B. subtilis* strain was used to further determine the DGC or PDE activities. The c-di-GMP metabolic genes were cloned into the pXG101 plasmid and transformed into an engineered *B. subtilis* strain, which is devoid of c-di-GMP metabolic enzymes to obtain heterologous expression strains. Experimental procedures were performed as described previously (Gao et al., 2013, 2014). Bacterial swarming motility was used as a platform to identify activation of c-di-GMP metabolic enzymes. Bacteria were grown on LB agar plates with antibiotics. A positive single colony was cultivated overnight in 2 mL LB medium at 37°C. 5 μL overnight cultures were inoculated onto LB plates containing 0.5% agar and 1 mM IPTG and grown for 12 h at 37°C. The bacterial swarm diameters were subsequently measured. Each assay was performed in at least three independent replications.

*In vitro* assays: PDEs activities were tested using a colorimetric assay. The reaction mixtures, containing 20 μg purified target protein and 5 mM bis (*p*-nitrophenyl) phosphate (bis-*p*NPP) substrate, were incubated at 37°C for 2 h in assay buffer (50 mM Tris-HCl, pH 8.5, 1 mM MnCl_2_) (Bobrov et al., 2005). Proteins possessing PDE activity will degrade the colorless bis-*p*NPP into yellow *p*-nitrophenyl, which was detected at 410 nm by a spectrophotometer. The experiments were performed in three independent replications.

Determination of DGCs and PDEs activities by High Performance Liquid Chromatography (HPLC) was performed according to previously described methods (Yang et al., 2012). Briefly, the putative DGC activity was determined by mixing 25 μg of purified proteins with 100 μM GTP in a reaction buffer (75 mM Tris-HCl, pH 7.8; 250 mM NaCl; 25 mM KCl; and 10 mM MgCl_2)_ in a total volume of 1 mL. Reactions were incubated at 28°C for 1 h. The putative PDE activity was determined by mixing 20 μg of purified proteins with reaction buffer (50 mM Tris-HCl, pH 8.5; 5 mM MgCl_2_; 10 mM MnCl_2_; 0.5 mM EDTA; and 50 mM NaCl) containing 10 μM c-di-GMP (Biolog, Germany) or 10 μM pGpG (Biolog, Germany), in a total volume of 1 mL. Reactions were incubated at 28°C for 1 h. The reactions were stopped by heating the samples for 5 min at 95°C, and then cooled on ice. Precipitates were removed by centrifugation. The supernatants were injected into an extended C-18-T column (250 × 4.6 cm) (Agilent) and separated by reversed-phase HPLC at a flow rate of 1.0 mL/min, and a linear gradient from 0 to 20% acetonitrile in buffer A (10 mm triethylammonium acetate, pH 5.8) for 10 min. The eluted substrate and products were detected by absorbance at 254 nm. Experiments were performed in triplicate.

### Determination of intracellular c-di-GMP concentration

Nucleotides were extracted as described previously (Spangler et al., 2010). Briefly, cell pellets were re-suspended in ice-cold extraction solution (40% methanol and 40% acetonitrile in 0.1 N formic acid) for 30 min. The cell suspension was then heated to 95°C for 10 min. After cooling, the suspension was centrifuged at 20,000 g for 5 min in order to separate insoluble material from the extracted nucleotides. The supernatant was collected and then evaporated until dryness at 40°C under a gentle stream of nitrogen gas. The residue was re-suspended in 50 μl of water under vigorous vortexing and then analyzed by LC-MS on an Agilent 1,290 infinity liquid chromatograph (Agilent Technologies, Wilmington, DE) coupled with an Agilent 6,460 triple quadruple mass spectrometer. Separation of compounds was achieved using reverse-phase HPLC, with a C-18 column of particle size 3 μm (5.5 cm length and i.d. of 4.6 mm) at 25°C. The mass spectrometer parameters were as follows: capillary voltage: 5,500 V, gas temperature: 350°C, fragmentor voltage: 75 V, collision energy: 34 V, nebulizer gas: 6 psi, curtain gas: 15 psi. Chromatographic separation was conducted in binary gradient using 10 mM ammonium formate, 0.1% acetic acid in water as solvent A and 0.1% formic acid in methanol as solvent B. The injection volume was 20 μl and the flow rate was 0.3 mL/min throughout the chromatographic run. The gradient was the following: time 0, 1% solvent B; 1 min, 1% solvent B; 2.5 min, 20% solvent B; 4 min, 20% solvent B; 7 min, 65% solvent B; 7.5 min, 95% solvent B; 9 min, 95% solvent B; 9.01 min, 1% solvent B; 10 min, 1% solvent B. Each sample was compared with a standard curve of c-di-GMP (Biolog, Germany), which was resuspended in water to quantify the amount of nucleotides.

### Effect of temperature-shift on the level of c-di-GMP

Leptospires were grown at 28°C in EMJH medium under aerobic conditions to 10^8^ CFU/mL and transferred to grow at 37°C. After incubation for 4 h, samples were collected by centrifugation at 12,000 g at 4°C for 10 min and stored at −20°C for quantification of c-di-GMP using HPLC-MS/MS. In parallel, bacteria were collected, treated with Trizol at time-points of 1, 2, and 4 h and stored at −80°C before quantification by real-time PCR. Leptospires incubated in growth medium at 28°C were used as controls.

### Host cell lines and culture conditions

The murine macrophage-like cell line J774A.1 was purchased from the Cell Bank at the Institute of Cytobiology, Chinese Academy of Science, Shanghai, China. The cells were grown in RPMI 1640 medium (Invitrogen, Carlsbad, CA) supplemented with 10% (V/V) heat-inactivated fetal bovine serum (FBS, Gibco/Invitrogen, Carlsbad, CA) in a humidified 5% CO_2_ atmosphere at 37°C. When required, 100 U/mL Penicillin (Sigma, USA) and 100 μg/mL streptomycin (Sigma, USA) were added to the cell culture medium.

### Determination of the level of c-di-GMP in leptospires infected cells

Fresh leptospires were collected by centrifugation at 8,000 g for 15 min and washed three times with sterilized 0.01 M PBS buffer (pH 7.4). Leptospires pellets were resuspended in RPMI 1640 medium with 10% (V/V) heat-inactivated FBS. The number of leptospires in the medium was counted under a dark-field microscope with a Petroff-Hausser counting chamber (Fisher Science, USA). The cell monolayers were washed three times with sterilized 0.01 M PBS buffer (pH 7.4), and fresh medium without antibiotics was added. Cells were cultured for 12 h before infection. J774A.1 cells (1 × 10^8^) were seeded to culture flasks (Corning, USA), incubated in an atmosphere of 5% CO_2_ at 37°C and then infected with leptospires at a multiplicity of infection (MOI) of 100 for 4 h. Leptospires cultured under identical conditions, but without J774A.1 cells were used as control. The unadhered leptospires were collected by centrifugation at 12,000 g for 10 min at 4°C. Infected cell lines were treated with 1% Triton X-100 and then centrifuged at 1,000 g for 5 min at 4°C to remove J774A.1. The leptospires in the supernatants were centrifuged at 12,000 g for 10 min at 4°C. All harvested leptospires were stored at −20°C for quantification of c-di-GMP levels by HPLC-MS/MS.

### Determination of c-di-GMP metabolic genes of *Leptospira* in infected cells

In order to determine the expression of c-di-GMP metabolic genes during infection of host cells, J774A.1 cells (1 × 10^6^ per well) were seeded in 6-well culture plates (Corning, USA) and incubated in an atmosphere of 5% CO_2_ at 37°C for 24 h. The cell monolayers were washed three times with sterilized 0.01 M PBS buffer (pH 7.4) and cultivated with fresh medium without antibiotics. Cells were infected with leptospires at a MOI of100 and incubated for 1, 2, 4, or 8 h. The unadhered leptospires were collected after centrifugation at 12,000 g for 10 min at 4°C. Infected cell lines were treated with 1% Triton X-100 and then centrifuged at 1,000 g for 5 min at 4°C to remove cell debris. The leptospires in the supernatants were collected through centrifuging at 12,000 g for 10 min at 4°C. Leptospires cultured under identical conditions but without cells were used as controls.

### RNA extraction, reverse transcription, and real-time quantitative PCR

All harvested leptospires were treated with Trizol reagent (Invitrogen, Carlsbad, CA) and RNA was extracted according to the manufacturer's protocol. cDNA was synthesized by reverse transcription using the PrimeScript RT reagent Kit with gDNA Eraser (TaKaRa, Japan). Quantitative real-time PCR was carried out using the SYBR Premix Ex-taq^TM^ Kit (TakaRa, Japan) and run on a Roche LightCycler 480II (Roche, Germany). The primers used in the reactions are shown in Table S1. 16S rRNA of *L. interrogans* was used as the internal reference. Relative quantification of c-di-GMP metabolic genes was calculated by the comparative 2^−ΔΔCt^ method to identify the relative mRNA levels of the samples after the thermal shift assays or infection assay. To compare the expression levels of each individual c-di-GMP metabolic gene, the relative mRNA abundance was calculated using the 2^−ΔCt^ (ΔCt = Ct _targetgene_ − Ct_16sRNA_) method (Livak and Schmittgen, 2001) and each result was multiplied by 10^6^. Three independent replicates were carried out for each experiment.

### Statistical analysis

Data were presented as means ± standard deviation. Two-tailed Student's *t*-test was used to determine the significance of differences in bacterial c-di-GMP levels and RFI between control and experimental groups. *P* < 0.05 were considered to be significant difference.

## Results

### Domain analysis of predicted c-di-GMP metabolic genes in *L. interrogans* Lai strain 56601

**Figure 1:**
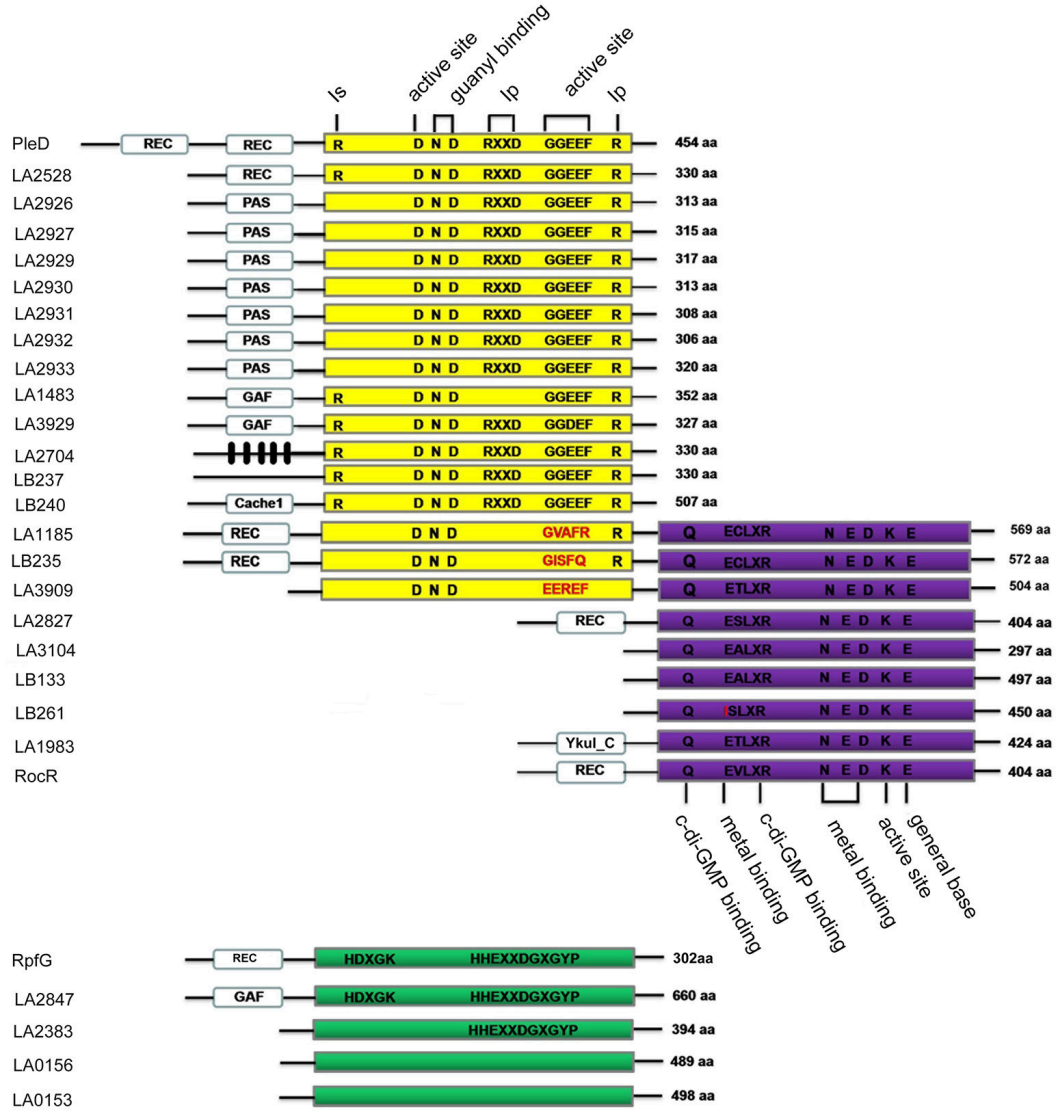
Schematic representation of the domain architecture of c-di-GMP metabolic enzymes of *L. interrogans* Lai strain 56601. Protein names are marked on the left and the number of amino acids is shown on the right. Yellow, purple, and green boxes represent GGDEF domains, EAL domains and HD-GYP domains, respectively. The GGDEF domain protein PleD from *C. Crescentus*, the EAL domain protein RocR from *P. Aeruginosa* and the HD-GYP domain protein RpfG from *X. campestris* are used as references to highlight amino acids important for enzymatic activity (Ryan et al., 2006; Wassmann et al., 2007; Rao et al., 2008). The putative conserved functional amino acid residues are shown in black (conserved) or red (mutation). Black boxes are predicted transmembrane regions. I_p_ is the primary inhibitory binding site for c-di-GMP, and I_s_ is the secondary inhibitory binding site for c-di-GMP. REC, PAS, Cache1, GAF, and YkuI_C sensory domains are indicated by white boxes.

In this study, 13 GGDEF domain proteins, 5 EAL domain proteins, 3 GGDEF-EAL domain proteins and 4 HD-GYP domain proteins were identified in *L. interrogans* Lai strain 56601 and predicted to be involved in the metabolism of c-di-GMP. These proteins were subsequently analyzed by multiple sequence alignment of the active motifs and conserved amino-acid residues (Figure 1). All the predicted GGDEF domain-containing proteins contained an intact active GG[D/E]EF motif. In addition, most of the GGDEF domain-containing proteins contained the RXXD inhibitory site, except for LA1483. All three predicted hybrid GGDEF-EAL domain proteins were identified to contain an intact EAL motif but an incomplete GGDEF motif. Except LB261, in which the E residue was substituted for an I residue, other predicted EAL domain-containing proteins contained an intact EXLXR motif. Among the four predicted HD-GYP domain proteins, only LA2847 contained both HD residues and the HHExxDGxxGYP motif. LA2383 also contained a HHExxDGxxGYP motif, but it lacked the conserved HD residues. Finally, LA0153 and LA0156 did not have either of the conserved HD residues or the HHExxDGxxGYP motif. Many GGDEF, EAL and HD-GYP domain-containing proteins are predicted to couple with sensory or regulatory domains, including the PAS, REC, GAF, YkuI_C, and Cache_1 domains. In addition, *L. interrogans* contained a cluster of seven genes that encode for the proteins LA2926~LA2933, all of which contained the PAS and GGDEF motifs (Figure S1A). Multiple sequences alignment showed high similarity in protein sequences among these seven PAS-GGDEF domain proteins (Figure S1B), suggesting that these genes may have derived from a common ancestor. Interestingly, we found that LA2828 which is adjacent to LA2827 (containing REC and EAL domains) is a gene encoding a putative histidine kinases (HK). These two genes constitute a two-component system (TCS), which may play essential roles in *Leptospira* to sense environmental signals and regulate cell functions.

### Phylogenetics of c-di-GMP metabolic proteins among *Leptospira* species

In order to investigate conservation and diversity of the c-di-GMP metabolism within the *Leptospira*, genetic information of fifteen *Leptospira* species was analyzed (Table 1). All analyzed *Leptospira* species contained multiple putative c-di-GMP metabolic genes, ranging from 17 genes in *L. borgpetersenii* to 25 genes in *L. interrogans*. The prevalence of c-di-GMP metabolic proteins among *Leptospira* species indicates that c-di-GMP may play an important role in regulating behavior in this genus. Seven sensory domains, including PAS, REC, GAF, Cache_1, 7TMR-DISM, PilZ, and YkuI_C were found to couple with GGDEF, EAL, or HD-GYP domains in the 15 analyzed *Leptospira* species. The GGDEF domain protein group, EAL domain protein group, HD-GYP domain and GGDEF-EAL domain protein group were specifically divided into 16 subgroups according to their coupled sensory domains (Table 1). Many PAS-GGDEF domain protein were located together in the four pathogenic strains of *Leptospira* (*L. interrogans, L. kirschneri, L. noguchii*, and *L. weilii*), similar as observed in *L. interrogans*, while only one PAS-GGDEF domain protein was found in each analyzed saprophytic stains or in another two pathogenic strain (*L. borgpetersenii* and *L. santarosai*) and none were found in intermediate pathogenic stains. The REC-GGDEF protein, which was predicted as a response regulator protein belonging to a two-component system, was present in 14 of the analyzed leptospiral species, except in *L. yanagawae*. GAF-GGDEF domain proteins were shared among all the 15 analyzed leptospiral species. Distribution of these proteins containing different domains in *Leptospira* suggested that these proteins may play important and conserved roles in c-di-GMP-dependent signal transduction in leptospiral species. We found that the Cache1-GGDEF domain containing proteins, 7TMR-DISM-GGDEF domain containing proteins and PilZ-GGDEF domain containing proteins exclusively existed in pathogenic, intermediate pathogenic or saprophytic species, respectively. In intermediate pathogenic *Leptospira* species, the majority of GGDEF domain proteins tended not to associate with any sensory domains, which was different from pathogenic species. Both REC-EAL domain proteins and YkuI_C-EAL domain proteins are shared among 13 of the analyzed *Leptospira* species. The PAS-GGDEF-EAL domain proteins were only shared within saprophytes, while the REC-GGDEF-EAL domain proteins were located in both pathogens and saprophytes but not in intermediate pathogenic species. Pathogenic and saprophytic species tended to contain more GGDEF-EAL domain proteins than intermediate pathogens. We found that the GAF-HD-GYP proteins are shared among all 15 selected species, indicating its evolutionary high conservation. The REC-HD-GYP proteins were shared among both intermediate and saprophytic species but were not present in pathogenic species. However, the majority of HD-GYP domain proteins were inclined to not couple with any sensory domains.

**Table 1:** Distribution of predicted c-di-GMP metabolic proteins in *Leptospira* species.

	**Strains**	**Pathogenicity**	**GGDEF only**	**PAS-GGDEF**	**REC-GGDEF**	**GAF-GGDEF**	**Cache1-GGDEF**	**7TMR-DISM-GGDEF**	**PilZ-GGDEF**	**Total number of GGDEF**	**EAL only**	**REC-EAL**	**YkuI_C-EAL**	**Total number of EAL**	**GGDEF-EAL only**	**PAS-GGDEF-EAL**	**REC-GGDEF-EAL**	**Total number of GGDEF-EAL**	**HD-GYP only**	**GAF-HD-GYP**	**REC-HDGYP**	**Total number of HD-GYP**	**Total number**
*L. borgpetersenii*	Hardjo-bovis str.L550	pathogenic	2	1	1	2	1			7	3	1		4	1		1	2	3	1		4	17
*L. santarosai*	Shermani str. LT 821	pathogenic	1	1	1	2	1			6	4	1		5	2		1	3	3	1		4	18
*L. interrogans*	Lai str. 56601	pathogenic	2	7	1	2	1			13	3	1	1	5	2		1	3	3	1		4	25
*L. kirschneri*	Grippotyphosa str. RM52	pathogenic	1	7	1	2	2			13	3	1	1	5	2		1	3	3	1		4	25
*L. noguchii*	Panama str. CZ214	pathogenic	1	7	1	2	2			13	3	1	1	5	2		1	3	3	1		4	25
*L. weilii*	Ranarum str. ICFT	pathogenic	1	8	1	2	2			14	2	1	1	4	2		1	3	3	1		4	25
*L. inadai*	Lyme str. 10	intermediate	4		1	1		1		7	2	2	2	6	1			1	6	1		7	21
*L. fainei*	Hurstbridge str. BUT 6	intermediate	5		1	1		1		8	2	2	2	6	1			1	4	1	1	6	21
*L. licerasiae*	Varillal str. VAR 010	intermediate	4		1	1		1		7	2	2	2	6	1			1	3	1	1	5	19
*L. broomii*	Hurstbridge str. 5399	intermediate	4		1	1		1		7	2	2	2	6	1			1	3	1	1	5	19
*L. wolffii*	Khorat str. Khorat-H2	intermediate	4		1	1		1		7	2	2	2	6	1			1	3	1	1	5	19
*L. wolbachii*	Codice str. CDC	saprophytic	1	1	1	1			1	5	3	2	2	7		3		3	2	1	1	4	19
*L. biflexa*	Patoc str. ‘Patoc 1	saprophytic	1	1	1	2			1	6	3		2	6	1	3	1	4	3	1		4	20
*L. meyeri*	Hardjo str. Went 5	saprophytic	1	1	1	2			1	6	3	1	1	5	1	3	1	5	3	1	1	5	21
*L. yanagawae*	Saopaulo str. Sao	saprophytic	2	1		2			1	6	3		2	5		3	1	4	3	1	1	5	20

**Figure 2:**
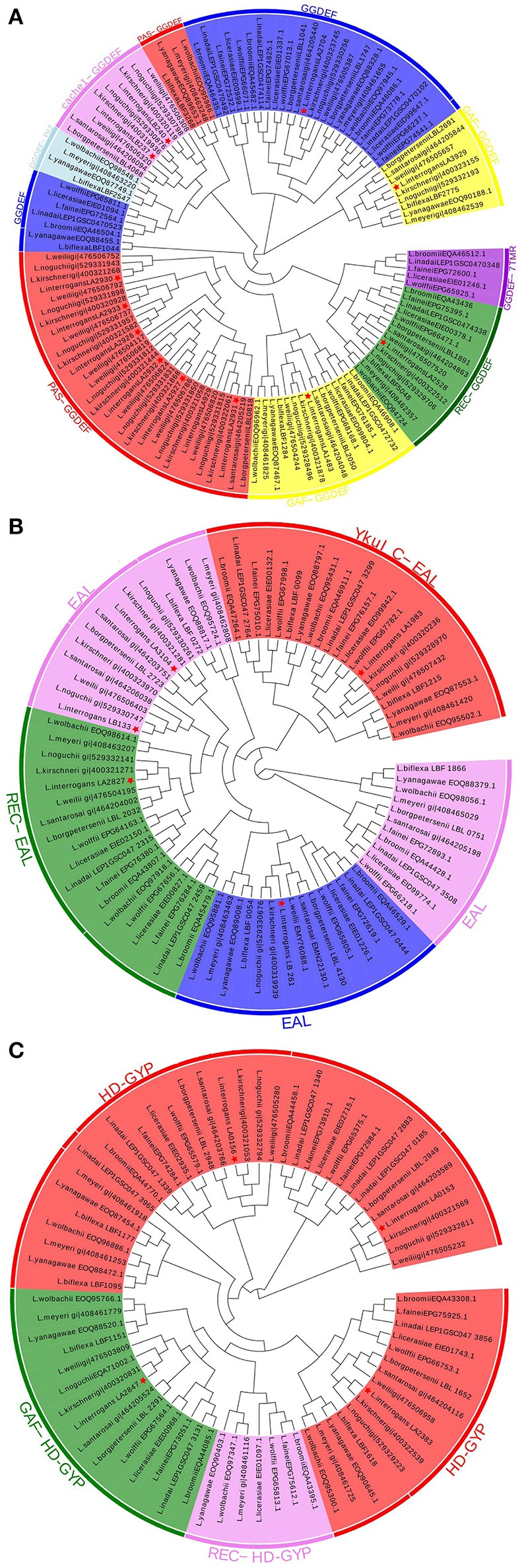
Conserved and unique C-di-GMP metabolic enzymes are widely distributed among pathogenic and saprophytic leptospiral species. Graphic representation of phylogenetic trees from 15 leptospiral species generated with aligned sequence of **(A)** GGDEF, **(B)** EAL, and **(C)** HD-GYP containing proteins. Protein architectures were written outside of circles, identical protein structures are marked by same colors. Proteins from *L. interrogans* are indicated by red asterisks.

To better understand the evolutionary relationships of c-di-GMP metabolic proteins among *Leptospira* species, phylogenetic trees were constructed based on the sequences of putative c-di-GMP metabolic proteins. As shown in Figure 2, c-di-GMP metabolic proteins belonging to species from the same pathogenicity group tended to cluster together, which is consistent with their genetic relatedness. These putative DGC or PDE proteins, which are from the common species, are almost separated to different clusters. All the PAS-GGDEF domain proteins from both pathogenic and saprophytic species formed two distinct clusters, suggesting that PAS-GGDEF domain proteins from pathogenic and saprophytic species have diverged (Figure 2A in red color). The proteins containing REC-GGDEF domains (Figure 2A in green color), YkuI_C-EAL domains (Figure 2B in red color), or GAF-HD-GYP domains (Figure 2C in green color), which were shared among almost all analyzed *Leptospira* species, were clustered together, suggesting that these DGC proteins showed high evolutionary conservation among *Leptospira*. Not only pathogenic, but also saprophytic species contain two GAF-GGDEF domain proteins. However, the two GAF-GGDEF domain proteins were distributed to separate phylogenetic clusters, implying that they have been derived from two distinct ancestors and therefore they might play different roles in c-di-GMP signaling pathways.

### Determination of DGC and PDE activities *in vivo*

**Figure 3:**
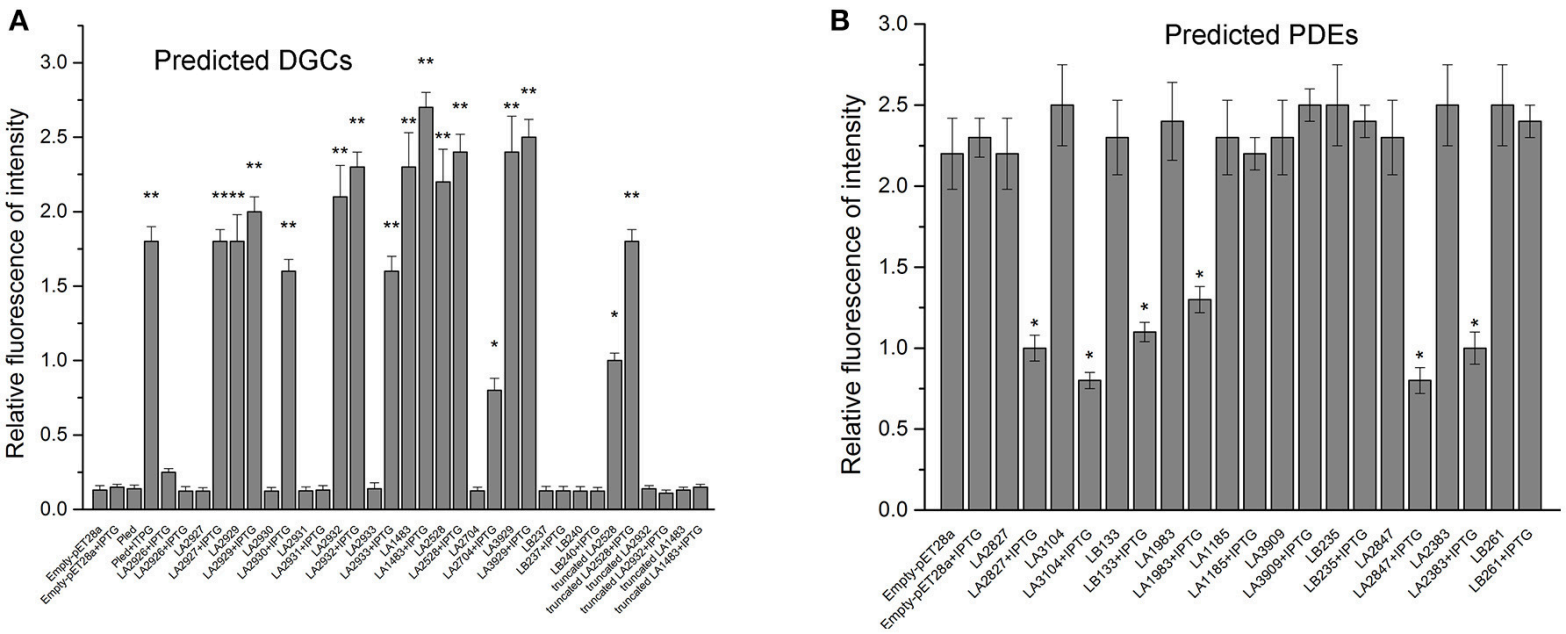
Multiple DGCs **(A)** and PDEs **(B)** from *L. interrogans* affect c-di-GMP accumulation in heterologous hosts. The RFI was defined as the ratio between the fluorescence intensities of TurboRFP at 574 nm and AmCyan at 489 nm and shows a linear correlation with intracellular c-di-GMP levels. Fluorescence intensity of DGCs and PDEs report strains were detected by Spectramax M5. Report strains culture, induction, photography and measurement of RFI were carried out as described in material section. Empty-pET-28a and pET-28a-pleD were used as negative control and positive control, respectively. Error bars represent the mean ± SD of three independent replicates. *P*-values were calculated by the two-tailed Student's *t*-test. ^*^p < 0.05, ^**^p < 0.01.

Although most of the c-di-GMP metabolic proteins contain active motif that are complete and are predicted to possess DGC or PDE activity, their specific enzymatic activities have not yet been experimentally validated. Therefore, enzymatic activities of all putative DGCs and PDEs were determined by heterologous co-expression with dual-fluorescence reporter plasmids in *E. coli* BL21 (DE3) (Zhou et al., 2016). Nine of recombinant reporter strains turned red before (LA2929, LA2932, LA1483, LA3929, and LA2528) or after (LA2927, LA2930, LA2933, and LA2704) induction with 1.0 mM IPTG (Figure S2A), indicating that these proteins display DGC activity. Another four putative DGCs (LA2926, LA2931, LB240, and LB237) did not display clear enzymatic activity, even though they were predicted to contain intact GGDEF motifs. Six reporter strains (LA2827, LA3104, LA1983, LA2847, LA2383, and LB133) shifted from dark to light red after induction with 1.0 mM IPTG (Figure S2B), indicating they display PDE activity. However, this shift is difficult to distinguish in bright field microscopy. Therefore, the RFI were determined to quantify shifts more accurately (Figure 3). Determination of The RFI values were in agreement with bright field microscopy. The nine recombinant reporter strains showing DGC activity exhibited a 10–20 fold increase in RFI values (Figure 3A), while the six recombinant reporter strains showing PDE activity exhibited a significant reduction in RFI values (Figure 3B).

Finally, to confirm enzymatic activity, LA1483, LA2528, and LB237 were expressed in an engineered *Bacillus subtilis* strain, which is deficient in its c-di-GMP metabolic enzymes (Gao et al., 2013). Assessment of enzymatic activity was carried out by observing motility changes. Expression of LA1483 and LA2528 in *Bacillus subtilis* resulted in inhibition of motility, while for LB237 no inhibition of motility was observed (Figure S3). These results were consistent with the dual-fluorescence reporter assays.

### Determination of putative DGC and PDE activities *in vitro*

**Figure 4:**
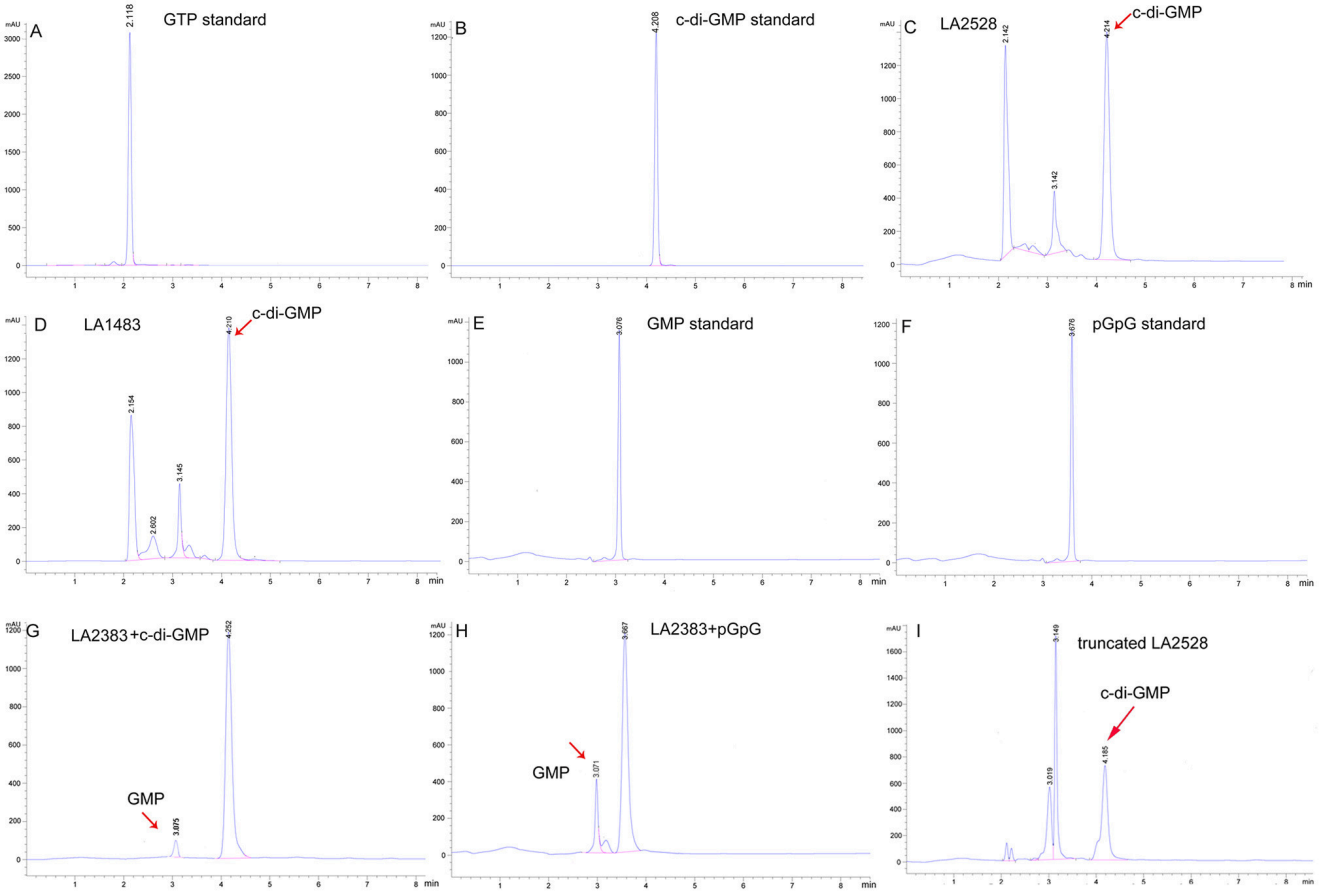
Selected c-di-GMP metabolic proteins from *L. interrogans* showed DGC and PDE activity *in vitro*. Enzyme reactions were performed as described in material sections. GTP was used as substrate in DGC activity assays. c-di-GMP or pGpG was used as substrate in PDE activity assays. **(A)** The retention time of GTP standard is 2.1 min; **(B)** The retention of c-di-GMP standard is 4.2 min; **(C)** LA2528 reaction product; **(D)** LA1483 reaction product; **(E)** The retention time of GMP standard is 3.0 min; **(F)** The retention time of pGpG standard is 3.6 min; **(G)** LA2383 reaction product with substrate c-di-GMP; **(H)**:LA2383 reaction product with substrate pGpG; **(I)** Truncated LA2528 reaction product. Peaks represent retention times. Red arrows represent reaction products.

The golden standards for determination of DGC or PDE activity are *in vitro* HPLC or LC-MS/MS analysis of c-di-GMP, GMP, or pGpG, an intermediate of c-di-GMP degradation (Christen et al., 2005; Bharati et al., 2012). LA2528 and LA1483, showing DGC enzymatic activities, and LA2383, showing PDE activity, were selected for *in vitro* HPLC analysis. HPLC results showed that both LA2528 and LA1483 display *in vitro* DGC activity, given that they are able to utilize GTP to produce c-di-GMP (Figures 4A–D). To determine the PDE activity of LA2383, bis (*p*-nitrophenyl) phosphate was used, which is a specific substrate for phosphodiesterases. LA2383 was able to degrade this substrate and produce a significant amount of *p*-nitrophenol compared with controls (Figure S4). PDE activity of LA2383 was further determined by HPLC using c-di-GMP and pGpG as substrates. LA2383 was able to hydrolyze both c-di-GMP and pGpG *in vitro* and produce GMP (Figures 4B,E–H).

### PAS and GAF sensory domains affect DGC activity

Many c-di-GMP metabolic proteins of *L. interrogans* contain sensory domains. Recombinant expression vectors were constructed encoding truncated LA2528 (no REC domain), LA2932 (no PAS domain) and LA1483 (no GAF domain). DGC activity of these truncated proteins was determined by fluorescence reporter assays *in vivo* and HPLC *in vitro*. Compared with the full-length proteins, truncated LA2932 and LA1483 lost their enzymatic activity, while truncated LA2528 still showed strong activity since the reporter strain turned red (Figure S2C and Figure 4I).

### High temperature decreases intracellular c-di-GMP levels in *L. interrogans*

**Figure 5:**
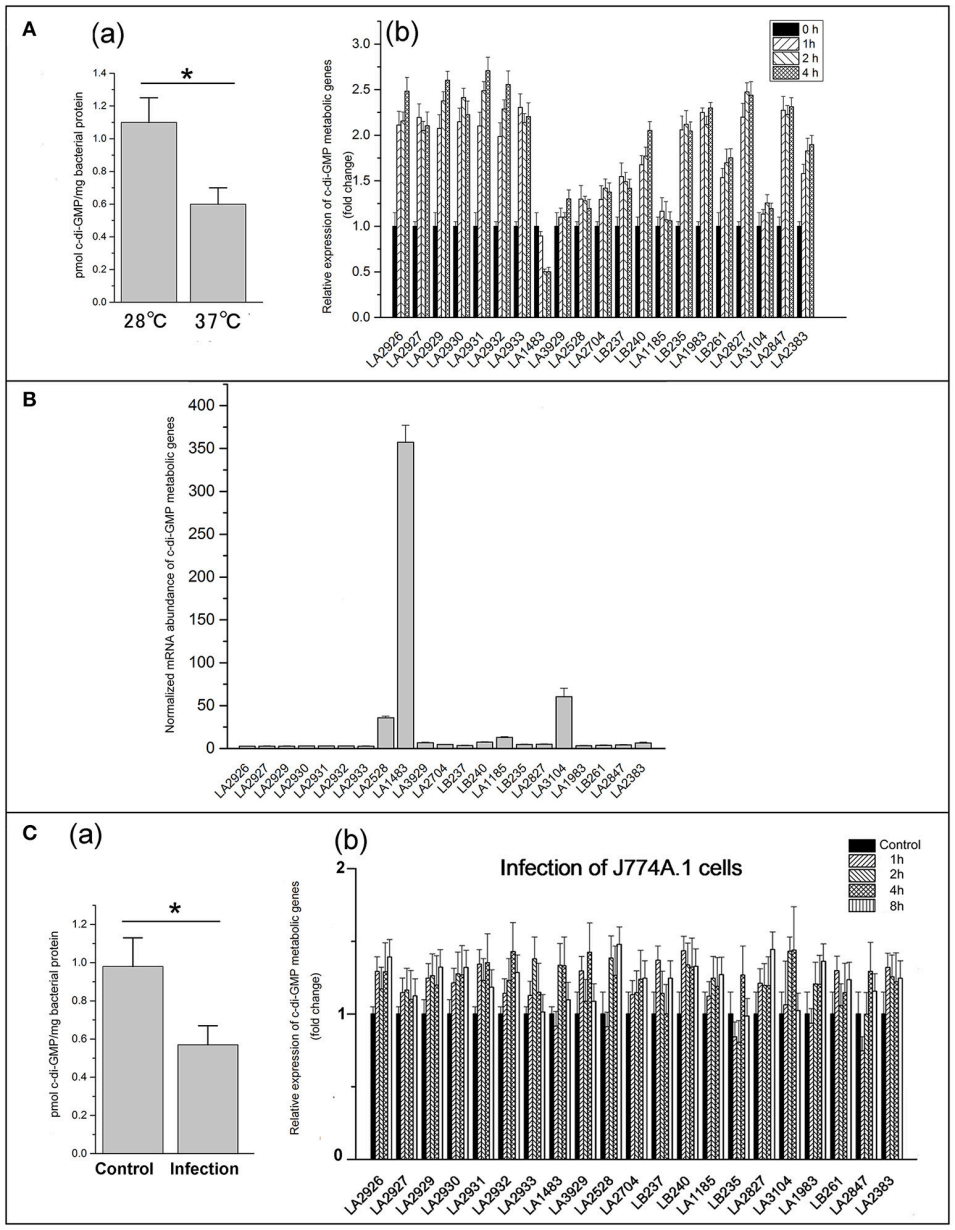
C-di-GMP levels and the expression of c-di-GMP metabolic genes varies in response to environmental changes. **(A)** Effect of temperature-shift on c-di-GMP levels **(a)** and expression of c-di-GMP metabolic genes **(b)**; **(B)** Normalized mRNA abundance of each c-di-GMP metabolic genes in *L. interrogans*; C: Determination of c-di-GPM levels **(a)** and expression of c-di-GMP metabolic genes **(b)** in *L. interrogans* during infection of mouse J774A.1 cells. The expression of c-di-GMP metabolic gene was measured by real-time PCR. The concentration of c-di-GMP was measured by HPLC-MS/MS. Error bars indicate standard deviations of three biological replicates. *P*-values were calculated by the two-tailed Student's *t*-test, ^*^p < 0.05.

Leptospires exhibit optimum growth *in vitro* at 28~30°C, but they are able to grow at 37°C when infecting a host (Cameron, 2015). To determine whether temperature affects cellular c-di-GMP levels, *L. interrogans* was cultivated in EMJH medium at 28°C and subsequently shifted to 37°C for 4 h. Quantification of c-di-GMP levels revealed that c-di-GMP levels decreased significantly after shifting from 28 to 37°C (Figure 5Aa). Expression of c-di-GMP metabolic genes of *L. interrogans* was subsequently determined by quantitative real-time PCR. As shown in Figure 5Ab, seven genes encoding DGCs containing a PAS-GGDEF domain were 2.5 fold upregulated after the temperature shift from 28 to 37°C. Similarly, four genes encoding active PDE proteins (LA2847, LA2383, LA2827, and LA1983) were upregulated 1.5–2.5 fold. Expression levels of the other DGC- and PDE-encoding genes were unaffected by the temperature shift. Only the LA1483 gene encoding active DGC protein exhibited light downregulated.

### Comparison of relative expression levels of c-di-GMP metabolic genes in *L. interrogans*

Although most DGCs and PDEs displayed enzymatic activities and differential expression during temperature-shift assay, their actual contribution to c-di-GMP levels in *L. interrogans* might be different. Therefore, we determined the relative expression levels of each c-di-GMP metabolic gene to estimate the relative contribution to the overall c-di-GMP levels (Figure 5B). Interestingly, the GAF-GGDEF domain-containing DGC LA1483 showed 7 to 100-fold higher expression levels compared with the other genes. Importantly, most of the c-di-GMP metabolic genes showed very low expression levels compared with LA1483 and only the REC-GGDEF domain-containing DGC LA2528 and EAL domain-containing PDE LA3104 exhibited relative high expression. Therefore, it appears that the overall c-di-GMP levels in *L. interrogans* are predominantly dependent on the activity of two DGCs and one PDE.

### Infection of host cells decreases intracellular c-di-GMP levels in *L. interrogans*

In order to determine whether the intracellular c-di-GMP levels are fluctuating during infection of host cells, mouse J774A.1 cells were infected with *L. interrogans* for 4 h. HPLC analysis revealed that intracellular c-di-GMP levels were significantly decreased during infection of mouse J774A.1 cells compared to control (Figure 5Ca). Further analysis of expression levels of c-di-GMP metabolic genes during the infection revealed that all the detectable c-di-GMP metabolic genes were slightly up-regulated but without significant variations during the infection of the mouse J774A.1 macrophages (Figure 5Cb).

## Discussion

Pathogenic species of *Leptospira* are the causative agents of leptospirosis, a widespread zoonotic disease. Understanding of the pathogenicity mechanisms of *Leptospira* species is still at an early stage due to the limited number of genetic tools available for this microorganism. C-di-GMP is as an important secondary messenger that regulates a variety of bacterial behaviors, including biofilm formation, motility, and virulence (Römling et al., 2013). However, so far research on c-di-GMP has been very limited in *Leptospira*. Although, recently a paper published by da Costa Vasconcelos and his colleagues has reported on a cAMP-dependent diguanylate cyclase from *L. interrogans* serovar Copenhageni (da Costa Vasconcelos et al., 2017).

In this study, we identified 25 proteins which were predicted to be involved in c-di-GMP metabolism in *L. interrogans* Lai 56601. The high number of c-di-GMP metabolic proteins implied that, as a second messenger, c-di-GMP may play important roles in *L. interrogans*. Most of the GGDEF and EAL domains were able to fuse to various sensory domains. These proteins could act independently from one another or be spatially and temporally regulated through sensing of various environmental or endogenous stimuli, indicating that they are might not be redundant (Römling et al., 2013). Interestingly, we found that LA2827, a response regulator containing REC and EAL domains, is located adjacent to LA2828, which is a histidine kinases containing HisKA and HATPase_c domains. Therefore, they constitute a typical two-component system involved in regulating c-di-GMP metabolism. In *Xanthomonas oryzae* pv. *oryzae*, Yang and his colleagues identified a novel two-component system, PdeK/PdeR, which regulates c-di-GMP turnover. PdeR was identified as a response regulator containing REC, GGDEF, and EAL domains and PdeR was shown to be a histidine kinases. The PdeK/PdeR TCS contributed to the virulence of *X. oryzae* pv. *oryzae* in rice (Yang et al., 2012). Therefore, we speculate that the LA2827/LA2828 TCS might also be required for the pathogenicity of *L. interrogans*.

We showed that most putative DGCs and PDEs of *L. interrogans* possessed enzymatic activities. However, four GGDEF domain proteins, which all contained conserved amino acid residues essential for synthesis of c-di-GMP, were proven to be inactive under our experimental conditions. These proteins were predicted to contain a RXXD I-site, which has previously been shown to bind c-di-GMP (Christen et al., 2006; Duerig et al., 2009). Furthermore, our analysis showed that three hybrid GGDEF-EAL domain proteins contained intact EAL conserved motifs and incomplete GGDEF domains, which therefore should possess PDE activity. However, in our experiments these three proteins showed neither DGC nor PDE activity. The failure to affect c-di-GMP levels is most likely not due to instability in *E. coli*, since they were successfully expressed and produced in strain BL21 (Figure S5). However, for *Pseudomonas aeruginosa* it has been shown that PelD, which is a degenerate GGDEF domain protein, acts as a c-di-GMP receptor that regulates biofilm formation (Whitney et al., 2012). Similarly, Filp, a degenerate EAL-GGDEF domain protein, regulates virulence in *X. oryzae* pv.*oryzae* through functioning as a cdi-GMP receptor (Yang et al., 2014). Therefore, we speculated that inactive GGDEF and EAL domain proteins may function as c-di-GMP receptors to regulate *L. interrogans* behavior. Interestingly, LA2383 contains an intact HHExxDGxxGYP motif but lacks HD amino acid residues, which have been shown to be essential to PDE activity (Römling et al., 2013; Bellini et al., 2014). However, LA2328 was still able to reduce c-di-GMP *in vivo* and *in vitro*. EAL domain-containing enzymes only hydrolyze c-di-GMP to pGpG (Christen et al., 2005), while pGpG is a nanoRNA molecule that can regulate gene expression (Nickels, 2012). LA2383 hydrolyzes c-di-GMP and pGpG into GMP, which results in decreased levels of c-di-GMP and pGpG and may help the leptospire respond to the niche changes rapidly.

PAS domains are found in all kingdoms of life and can sense environmental and nutritional signals, such as light, oxygen, metabolites or redox potential, through detection of co-factors such as heme (Taylor and Zhulin, 1999). Interestingly, seven genes encoding PAS-GGDEF domain proteins are located in a single operon in the genome of *L. interrogans* and showed high similarity in their sequences. In our previous work, we have found that during a *Leptospira* infection, mRNA levels of hemolysins were increased significantly (Xue et al., 2010). Hemolysins are able to destroy red blood cells, resulting in release of heme and ROS (data not shown). So, we speculate that leptospires with more PAS-GGDEF domain proteins may adapt better to fluctuating environments during an infection. When the PAS domain in LA2932 was deleted, its DGC activity was reduced, suggesting that PAS domains are required for DGC activity. The GAF domain is found in all three kingdoms of life as well and can bind a variety of ligands, including formate, heme, bilin and cNMP (Anantharaman et al., 2001; Sousa et al., 2007; Vos et al., 2012; Biswas et al., 2015). In this study, we observed that deletion of GAF from LA1483 resulted in loss of DGC activity, suggesting that the GAF domain is required for the DGC activity of LA1483. In *L. interrogans* serovar Copenhageni, Vasconcelos and his colleagues reported a GAF and GGDEF domain-containing protein, corresponding to LA3929, for which the DGC activity could be significantly enhanced by binding of cAMP with the GAF domain (da Costa Vasconcelos et al., 2017). Both cAMP and c-di-GMP are secondary messengers in bacteria. The GAF and GGDEF domain-containing protein may play an important role in integrating cAMP and c-di-GMP signaling in *L. interrogans*.

REC is as a two-component signaling receiver domain, which has been reported to modulate protein-protein interaction (Chen et al., 2015; Su et al., 2015; Xu et al., 2016). However, in this study we found that truncated LA2528 retained its ability to synthesize c-di-GMP but not as strong as full-length protein, suggesting that the REC domain has a little effect on DGC activity.

During infection of host animals, Leptospires must endure fluctuations in temperature from 28°C (environment) to 37°C (host) or even 39°C (fever), so temperature represents an important external stress, which may therefore influence virulence and viability of pathogenic *Leptospira*. Since c-di-GMP was reported as a secondary messenger that regulates bacterial behavior by sensing external changing environments, we predicted that c-di-GMP levels in *Leptospira* may be affected by temperature. In this study, we found that intracellular c-di-GMP levels were down-regulated in response to a temperature-shift from 28 to 37°C, and that mRNA levels of four genes encoding PDEs were up-regulated during the temperature shift from 28 to 37°C. These four proteins were proven to possess PDE activities, so their induction might explain the reduction of c-di-GMP concentrations. Only LA1483, a GAF-GGDEF domain protein, was down-regulated after the temperature shift to 37°C, which may decrease the production of c-di-GMP. However, we also observed that the mRNA levels of seven GGDEF-containing proteins were up-regulated, and five of them proved to possess DGC activity, which should result in increased c-di-GMP levels. However, correlation of expression of c-di-GMP metabolic genes with actual c-di-GMP levels is complicated and dependent on the individual contribution of each gene. However, while most of the c-di-GMP metabolic genes showed low overall mRNA levels, mRNA levels of LA1483 were relatively high, indicating that it might have a significant impact on the overall c-di-GMP levels. In *Yersinia pestis*, which contains a multitude of GGDEF domain proteins, specifically HmsT is responsible for 75~80% of the cellular c-di-GMP levels (Bobrov et al., 2011). Therefore, we predict that LA1483 might have a dominant effect on the c-di-GMP levels of *Leptospira* during the temperature shift and be responsible for the decreased c-di-GMP levels. Townsley and his colleagues similarly found that during a temperature downshift from 37 to 15°C in *V. cholera*, expression levels of several genes encoding DGCs were increased. This led to an increase of intracellular c-di-GMP levels and resulted in the formation of biofilms (Townsley and Yildiz, 2015). Temperature affecting GGDEF/EAL gene expression was also found in *E. coli*, for which most GGDEF/EAL genes were up-regulated at 28°C compared with 37°C (Sommerfeldt et al., 2009). Previous studies have reported that many *L. interrogans* genes involved in motility, chemotaxis and two-component signal transduction systems were differentially expressed upon a temperature shift from 25 to 37°C (Nally et al., 2001; Lo et al., 2006; Qin et al., 2006; Oliveira et al., 2010). Due to the high level of expression of LA1483 compared to other c-di-GMP metabolic genes, and its pattern of expression during thermal shift, we hypothesized that it may play an important role in controlling c-di-GMP homeostasis in *L. interrogans*. To further investigate the role of c-di-GMP signaling in *L. interrogans* we attempted a knockout of LA1483 but failed to obtain the desired mutation. We currently do not know if this failure was due to limited availability of efficient gene editing tools in *L. interrogans* or because the absence of this gene could affect the fitness of this organism under the conditions used to retrieve the knock out. The use of inhibitors of DGC activity might prove useful to address the role of c-di-GMP signaling in *L. interrogans* and other organisms that are recalcitrant to genetic modification (Ching et al., 2010; Sambanthamoorthy et al., 2012, 2014; Zhou et al., 2013; Fernicola et al., 2015).

During an infection, the growth environment, such as oxygen levels, acidity and hypertonicity, of *L. interrogans* changes. DGCs and PDEs frequently harbor sensory domains, which potentially modulate DGC and PDE activity by sensing environmental signals and result in variation of intracellular c-di-GMP levels. In this study, we found that infection of J774A.1 cells decreased intracellular c-di-GMP levels in *L. interrogans*. Real-time PCR results showed all c-di-GMP metabolic genes were unaltered during infection of J774A.1 cells, suggesting that the reduction of c-di-GMP levels must be regulated by variation of DGCs or PDEs activities. In our previous study, we found that infection of J774A.1 cells resulted in induction of genes involved in chemotaxis and oxygen tolerance (Xue et al., 2010). In many bacterial species, it is well established that c-di-GMP levels regulate motility, which is evolutionary conserved (Römling et al., 2013). In addition, four putative c-di-GMP-binding PilZ domain proteins were found in the genome of *L. interrogans*. Therefore, it is reasonable to assume that c-di-GMP levels in *L. interrogans* regulate motility and pathogenicity through regulation of the expression of virulence factors. In addition, c-di-GMP has been shown to be an immunomodulatory trigger of the host immune response (Karaolis et al., 2007; Ogunniyi et al., 2008), while low c-di-GMP levels may contribute to immune evasion.

In summary, our results offer an important starting point for future functional studies of c-di-GMP signaling in leptospiral species.

## Author contributions

XL and JY designed the work. GX, LK, RC, YY, and QZ performed the laboratory work. GX wrote this manuscript. LX revised this manuscript.

### Conflict of interest statement

The authors declare that the research was conducted in the absence of any commercial or financial relationships that could be construed as a potential conflict of interest.
