# Supplementary figures and images for: Identification and Characterization of c-di-GMP Metabolic Enzymes of Leptospira interrogans and c-di-GMP Fluctuations After Thermal Shift and Infection
Source: Front Microbiol. 2018 Apr 20;9:764. doi: 10.3389/fmicb.2018.00764 (PMC5932348; doi:10.3389/fmicb.2018.00764)

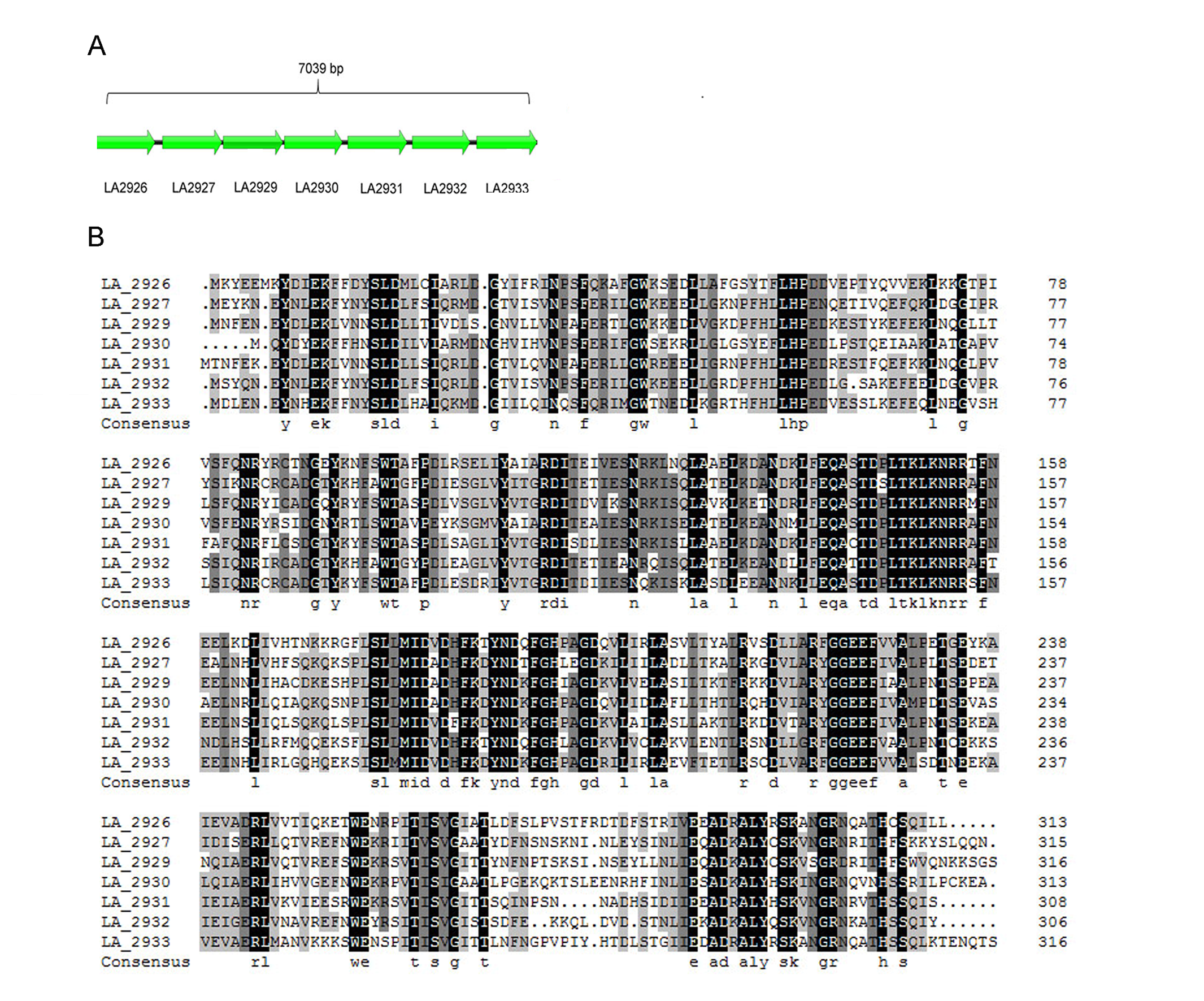

Supplement: Supplementary file 1 [file Image1.TIF]

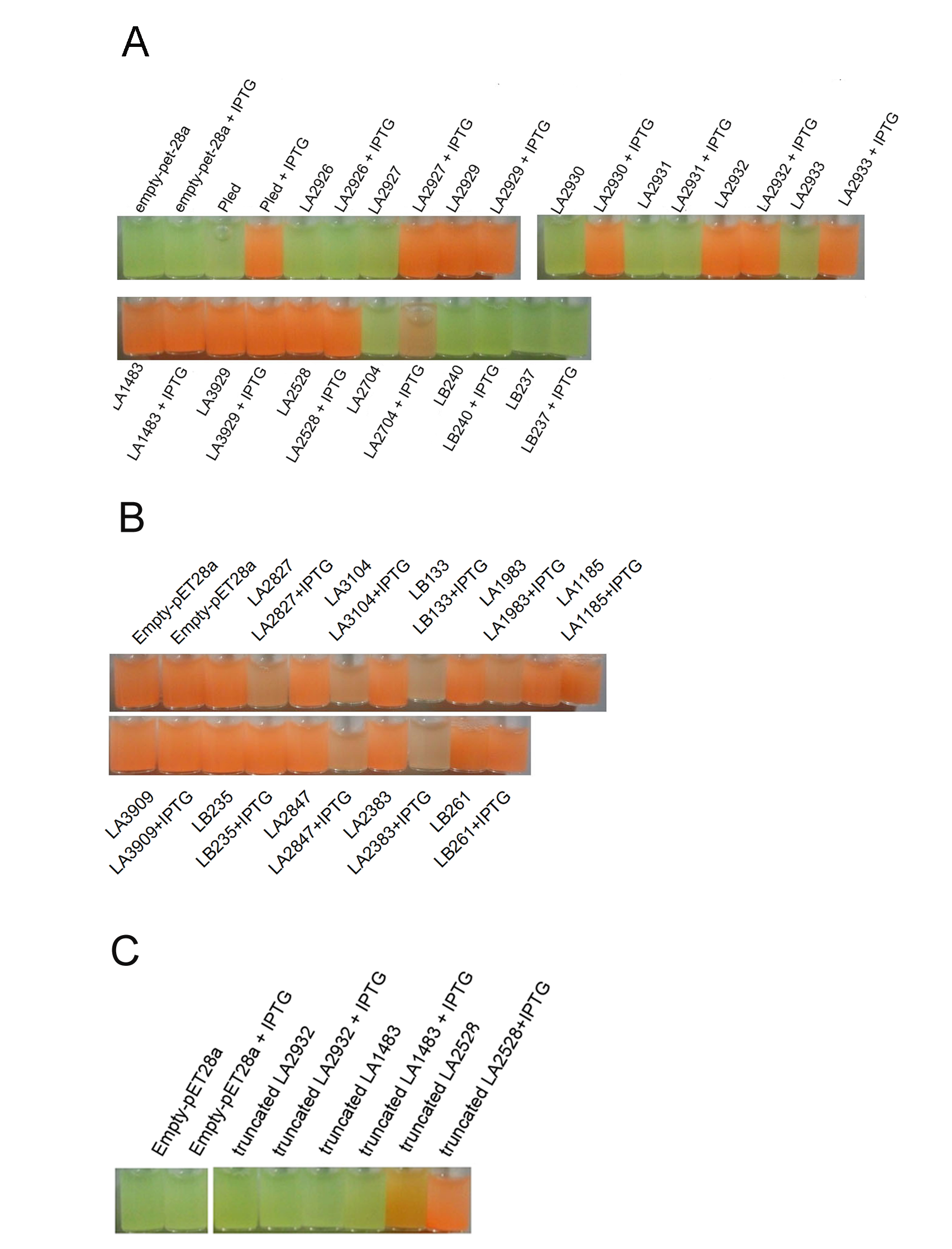

Supplement: Supplementary file 2 [file Image2.TIF]

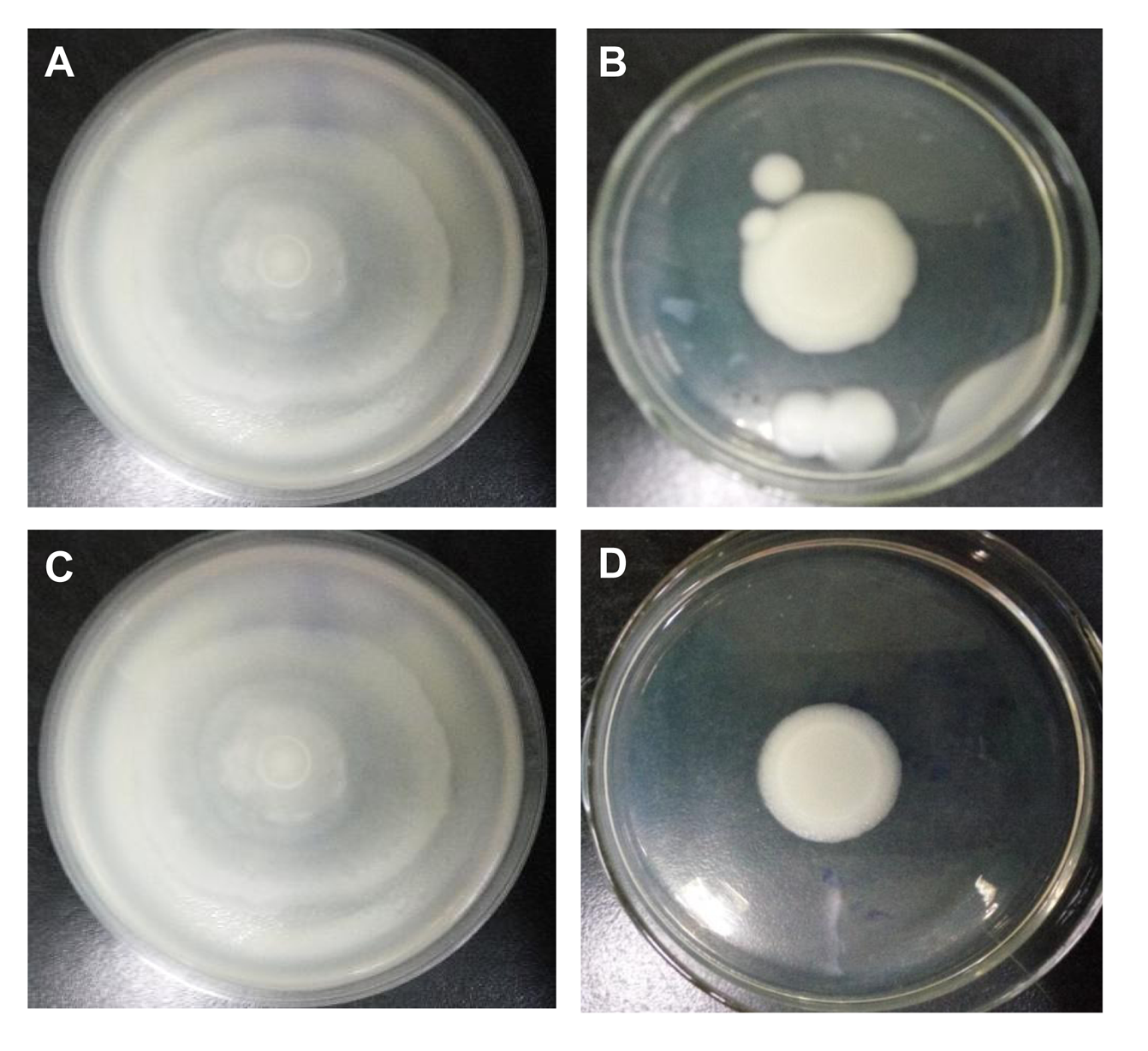

Supplement: Supplementary file 3 [file Image3.TIF]

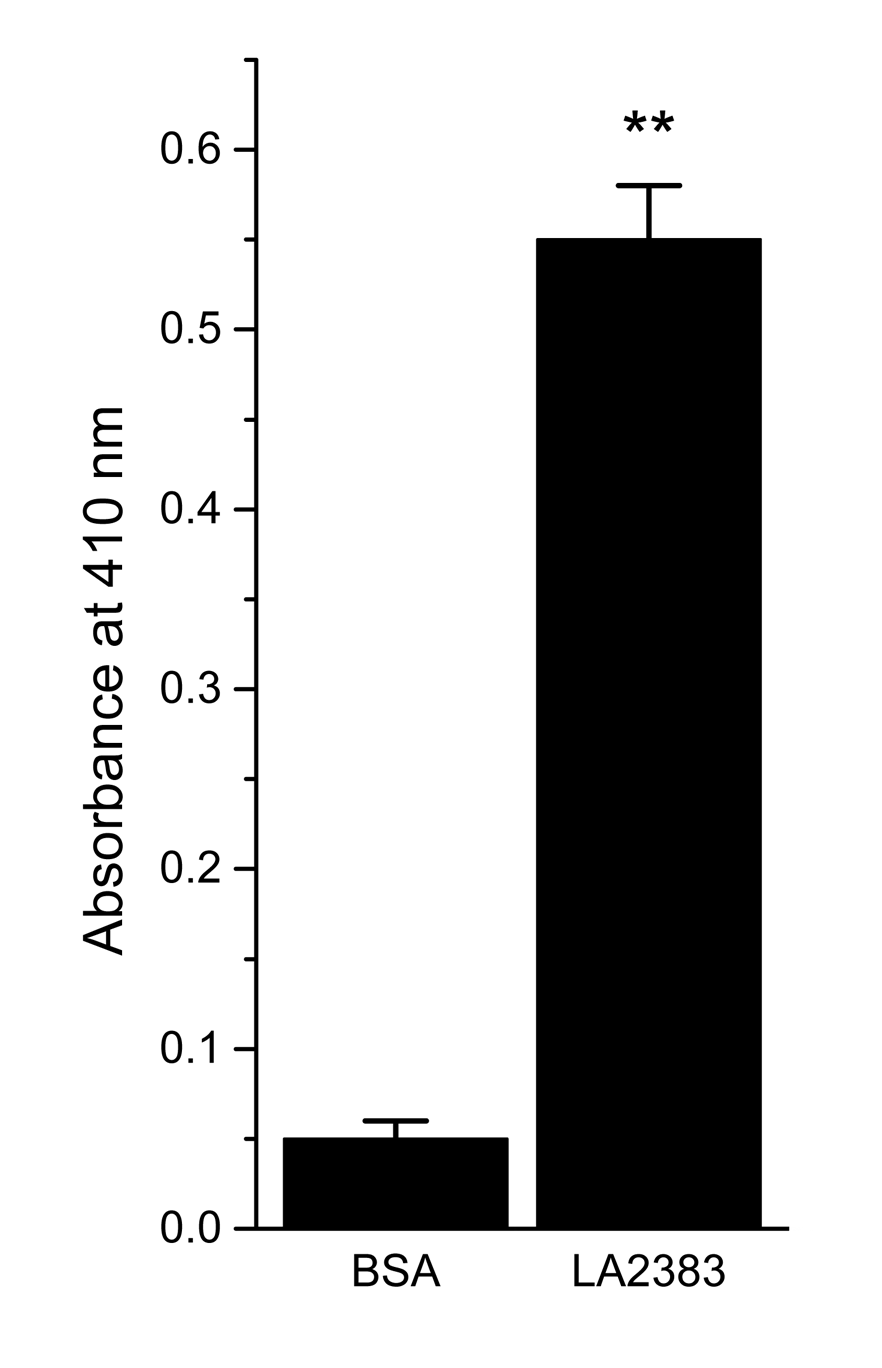

Supplement: Supplementary file 4 [file Image4.TIF]

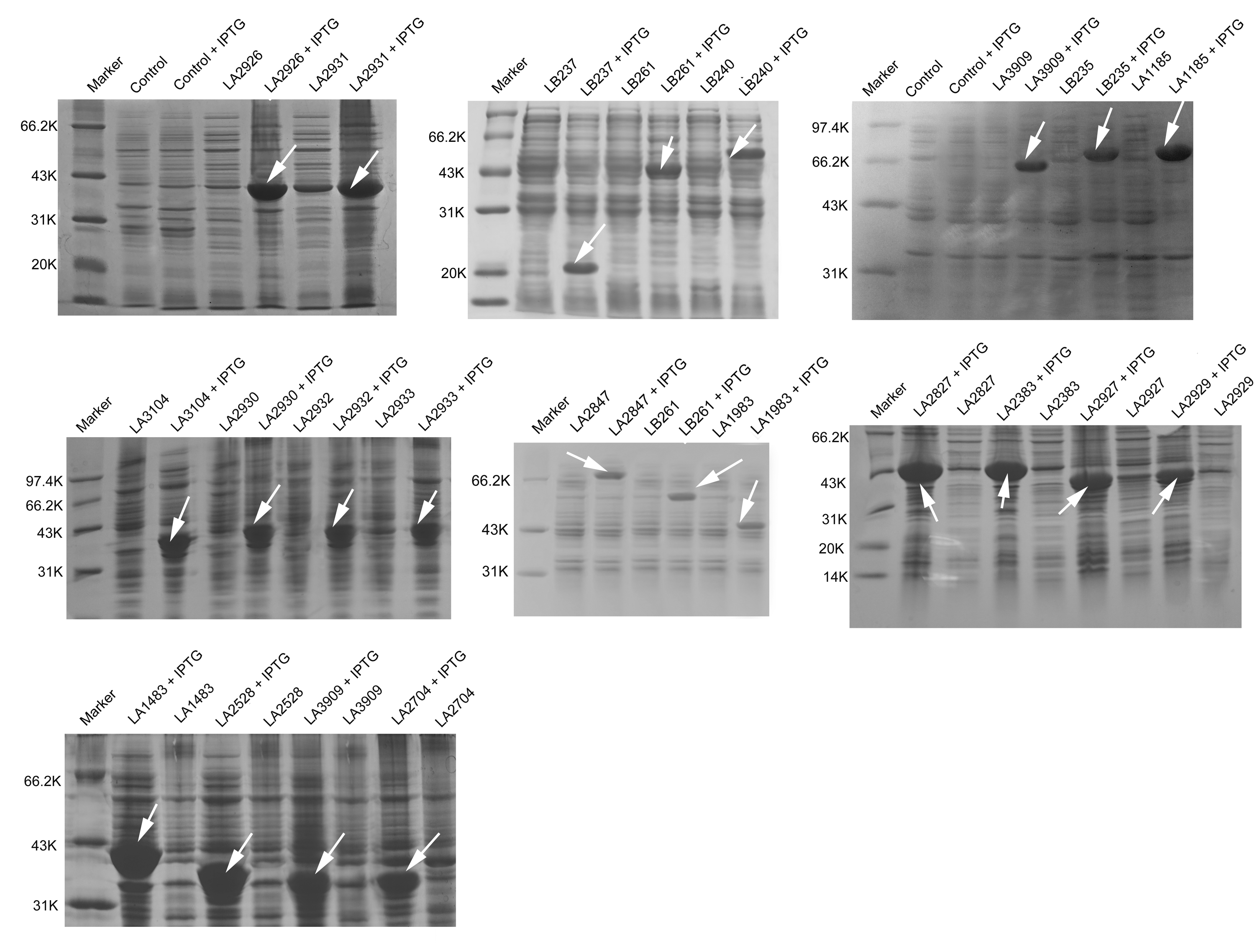

Supplement: Supplementary file 5 [file Image5.TIF]
