# Supplementary material for: Identification and Characterization of c-di-GMP Metabolic Enzymes of Leptospira interrogans and c-di-GMP Fluctuations After Thermal Shift and Infection
Source: Front Microbiol. 2018 Apr 20;9:764. doi: 10.3389/fmicb.2018.00764 (PMC5932348; doi:10.3389/fmicb.2018.00764)
Supplement: Supplementary file 6 [file Table1.DOCX]

***Supplementary Material***

**Metabolism and Possible Roles of c-di-GMP in the Survival and Pathogenesis of *Leptospira interrogans***

Guohui Xiao^1^, Liangliang Kong^1, 2^, Rongbo Che^1^, Yusi Yi^1^, Qinchao Zhang^1^, Jie Yan^1, 3*^and Xu’ai Lin^1, 3*^

^1^Department of Medical Microbiology and Parasitology, School of Medicine, Zhejiang University, China,

^2^Zhejiang Tianke High Technology Development CO. Ltd

^3^Basic Medical Microbiology Division, State Key Laboratory for Diagnosis and Treatment of Infectious Diseases, School of Medicine, Zhejiang University, China

* Corresponding authors

Jie Yan: Basic Medical Microbiology Division, State Key Laboratory for Diagnosis and Treatment of Infectious Diseases, School of Medicine, Zhejiang University, Hangzhou 310058, China, Tel: 86-571-88208296, E-mail address: med_bp@zju.edu.cn;

Xu’ai Lin: Department of Medical Microbiology and Parasitology, School of Medicine, Zhejiang University, 866Yuhangtang Road, Hangzhou 310058, China, Tel&Fax: 86-571-88208294, E-mail address: lxai122@zju.edu.cn.

TableS1 Primers used in this study

| Primer name | Primer sequences (5’ ~ 3’) | Purpose |
| --- | --- | --- |
| LA1483 | F: CCC**GAATTC**ATGATCAGTAAAGAAAACGA | plasmids construction |
|  | R: GGA**CTCGAG**CTAATTTTCTTTGTAACAAAC | plasmids construction |
| LA2926 | F: CCT**GGATCC**ATGAGGCTTTCTTTGGGAAAT | plasmids construction |
|  | R: GGT**CTCGAG**CTAAGAAGCAGCAGTAGTTC | plasmids construction |
| LA2927 | F: CCT**GGATCC**TTGGAATATAAAAACGAATAC | plasmids construction |
|  | R: GGT**CTCGAG**TTAATTTTGTTGTAAGGAGTAT | plasmids construction |
| LA2929 | F: CCT**GGATCC**ATGAATTTTGAAAACGAATAC | plasmids construction |
|  | R: GGT**CTCGAG**TTAGATAGATCCGCTCTTTTTATT | plasmids construction |
| LA2930 | F: CCT**GGATCC**ATGACGAATTTTGAAAAAGAATAC | plasmids construction |
|  | R: GGT**CTCGAG**TCAAGATATTTGGGAACTAT | plasmids construction |
| LA2931 | F: CCT**GATATC**ATGCAGTACGATTATGAAAAATTTT | plasmids construction |
|  | R: GGT**CTCGAG**TTACGCTTCTTTACAAGGAAGG | plasmids construction |
| LA2932 | F: CCT**GGATCC**ATGAGTTATCAAAACGAATAC | plasmids construction |
|  | R: GGT**CTCGAG**TTAATATATTTGAGAACTATGAG | plasmids construction |
| LA2933 | F: CCT**GATATC**ATGGATCTCGAAAACGAATATAAT | plasmids construction |
|  | R: GGT**CTCGAG**TCAATTTTTGCGAACGGACGTT | plasmids construction |
| LA3929 | F: CCT**GGATCC**ATGACCTTAAAAGACTCAAAACC | plasmids construction |
|  | R: GGT**CTCGAG**TCATCCTTTGGATGCATACATAG | plasmids construction |
| LA2528 | F: CCT**GGATCC** ATGGATTCCATTCTTATTTTAGAC | plasmids construction |
|  | R: GGT**CTCGAG**TTAATCATTTTGAGGATAAAATCGA | plasmids construction |
| LA2704 | F: CCT**GGATCC**ATGAGGCTTTCTTTGGGAAATAA | plasmids construction |
|  | R: GGT**CTCGAG**CTAAGAAGCAGCAGTAGTTCCC | plasmids construction |
| LB237 | F: CCT**GGATCC**ATGAACCAAGTATCTAAATTAATTC | plasmids construction |
|  | R: GGT**CTCGAG**TTATTTTAAACTCGATTGCAAAAAG | plasmids construction |
| LB133 | F: CCT**AAGCTT**ATGAATTTGGATCCAAAACTTAAAA | plasmids construction |
|  | R: GGT**CTCGAG**TTATTTTAAGAAATGATCTTGAAATT | plasmids construction |
| LB240 | F: CCT**GGATCC**ATGGTCTTAGCCGTTATCCCG | plasmids construction |
|  | R: GGT**CTCGAG**TTAAAACATTTCCGTTGTAGTATC | plasmids construction |
| LA3909 | F: CCT**GGATCC**GTGCTTTACGAAAAAATCATTCC | plasmids construction |
|  | R: GGT**CTCGAG**TTAAGTAGCGGGGATCGCC | plasmids construction |
| LA1185 | F: CCT**GGATCC**ATGGACGGAAATATAAATTCTC | plasmids construction |
|  | R: GGT**CTCGAG**TTAAACAGAAACTTGAGGAATCG | plasmids construction |
| LA1983 | F: CCT**GGATCC**ATGATCGGCATTTCTCCAAAAACG | plasmids construction |
|  | R: GGT**CTCGAG**CTAATATTCAAGAACTTGAGCA | plasmids construction |
| LA2827 | F: CCT**GGATCC**ATGAATCAGACCACATTACCC | plasmids construction |
|  | R: GGT**CTCGAG**TCAAGATTTAAAACCAGCTAAGA | plasmids construction |
| LA3104 | F: CCT**GGATCC**ATGAAAGAAGTTTTTGATTTACC | plasmids construction |
|  | R: CCT**GGATCC**TTAAACTAAAACGTTCCTAGATTG | plasmids construction |
| LA2847 | F: CCT**GGATCC**ATGAAATCGAAATTCCCTAAATAC | plasmids construction |
|  | R: CCT**GGATCC**CTAACGAAGGTAACCAGAGTAAT | plasmids construction |
| Truncated LA1483 | F: CCC**CATATG**GATATGATGACTGGACTC | plasmids construction |
|  | R: GGG**CTCGAG**CTAATTTTTAGCTGTATAAAG | plasmids construction |
| Truncated LA2528 | F: CCC**CATATG**CAAGAAGTCAACGCC | plasmids construction |
|  | R: GGG**CTCGAG**ATCATTTTGAGGATAAAATC | plasmids construction |
| Truncated LA2929 | F: CCC**CAT ATG**ATGAGTTATCAAAACGAATACA | plasmids construction |
|  | R: GGG**CTCGAG**TTAATATATTTGAGAACTATGAGT | plasmids construction |
| LA1483dgc | F: GGA**GCTAGC**ATCAGTAAAGAAAACGATCCC | plasmids construction |
|  | R: GGA**GCATGC**CTAATTTTCTTTGTAACAA | plasmids construction |
| LA2528dgc | F: GGA**GCTAGC**GATTCCATTCTTATTTTAGA | plasmids construction |
|  | R: GGA**GCATGC**TTAATCATTTTGAGGATAAA | plasmids construction |
| LB237dgc | F: GGA**GCTAGC**AACCAAGTATCTAAATTAATTC | plasmids construction |
|  | R:GGA**GCATGC**TTATTTTAAACTCGATTGCAAA | plasmids construction |
| LA2926Q | F: TCGCCTTCGGTTCTTATACATTC | Real time RT-PCR |
|  | R: TCATTTGCGTCTTTGAGTTCTGC | Real time RT-PCR |
| LA2927Q | F: CCGTCATTTCCGTAAATCCATCC | Real time RT-PCR |
|  | R: TTTGTACGTTCCGTCTGCACATC | Real time RT-PCR |
| LA2929Q | F: CCCTGACTTGGTGTCTGGTTTAG | Real time RT-PCR |
|  | R: GCAATATGTCCGAATTTGTCGTT | Real time RT-PCR |
| LA2930Q | F: AGGCTTAATTTATGTAACCGGAAGG | Real time RT-PCR |
|  | R: CGATCATCAACAAGGAAAGAGGG | Real time RT-PCR |
| LA2931Q | F: ACTGGCGCTCCGGTCGTATCTTT | Real time RT-PCR |
|  | R: AGGGTCTGTGCTCGCTTGTTCTA | Real time RT-PCR |
| LA2932Q | F: CTGTGATCTCCGTCAATCCCTCA | Real time RT-PCR |
|  | R: TCCGGTCCAAGCGAAATGTTTAT | Real time RT-PCR |
| LA2933Q | F: AATCCAGCCTAAAAGAATTTGAACA | Real time RT-PCR |
|  | R: TTTGATTCGACTCGATGATGTCAGT | Real time RT-PCR |
| LA1483Q | F: GAGAATATGGGTAGGGCTGTAAATG | Real time RT-PCR |
|  | R: CTCTATCCTCTTCTTGGACTTCGTT | Real time RT-PCR |
| LA3929Q | F: TGGAGTTTGATGCGTTACGATGA | Real time RT-PCR |
|  | R: GTTTGGACAACTGAACCGGCAAT | Real time RT-PCR |
| LA1185Q | F: GAATCTTCTACTGCCACAACTCATC | Real time RT-PCR |
|  | R: AATAAACTGCCCGAGTCTCCCATAA | Real time RT-PCR |
| LA3909Q | F: TAAACCTCAACCAAATGCTCAACCC | Real time RT-PCR |
|  | R: CGATCTGCTACTTTGACGTAAATGG | Real time RT-PCR |
| LB235Q | F: GCAAGATGTAAGTCAGAACAGATAG | Real time RT-PCR |
|  | R: CAGAATCCCAAGGTTTAGTAAGGTA | Real time RT-PCR |
| LA2528Q | F: ACTTTTGGCAAGAGTTCGTTCGG | Real time RT-PCR |
|  | R: CTTGAAGTTGATCGGTCAGGTTT | Real time RT-PCR |
| LA2704Q | F: ATCTGCTTTCTTCGCATTTCTGC | Real time RT-PCR |
|  | R: ATGACTACGGTTGTTCCGGTGAG | Real time RT-PCR |
| LB237Q | F: TGTGGTTTGTCGTTGGGGTGGAG | Real time RT-PCR |
|  | R: TGCTTGATACAACGCTTGGTCGG | Real time RT-PCR |
| LB240Q | F: CAACTCCTAAAACGAATCCTCAG | Real time RT-PCR |
|  | R: TCTACGCATTGCATCAAGACCTA | Real time RT-PCR |
| LA1983Q | F: GGATCATATCGAGGAAGAAGAGG | Real time RT-PCR |
|  | R: CCCAAATCGTCAATCGCTACTAAAA | Real time RT-PCR |
| LA3104Q | F: GCCCTAGCCAGGTTCCGTATCAA | Real time RT-PCR |
|  | R: ATTCGGCAGCCTGTGAACGATTT | Real time RT-PCR |
| LB133Q | F: TCCGAAGCGAATACGAGCCCTCT | Real time RT-PCR |
|  | R: ATGTTGCGATCAAATTGGAAGAA | Real time RT-PCR |
| LB261Q | F: AAAGCAGACGCTGTCATTCAACC | Real time RT-PCR |
|  | R: TCGAATTAAATCCGCATAACCAG | Real time RT-PCR |
| LA2383Q | F: ACGAACAACTCACCGATGAGGAA | Real time RT-PCR |
|  | R: CTGAAGGGCAACAATCGCAAGAT | Real time RT-PCR |
| LA2847Q | F: GTAGGGATTAGTCTTGCCGATGA | Real time RT-PCR |
|  | R: TAACCCGCAACACTGGATTTATT | Real time RT-PCR |
| LA2827Q | F: TTTTGAAGTTACGATTACCCACG | Real time RT-PCR |
|  | R: TATTGGATCGCAAGTGCTTCTGC | Real time RT-PCR |
| 16S | F: CTTTCGTCGCCTCAGCGTCAGT | Real time RT-PCR |
|  | R: CGCAGCCTGCACTTGAAACTA | Real time RT-PCR |

**Supplementary Figure legends**

**Figure S1. Layout of genes encoding PAS-GGDEF domain proteins cluster in the genomes of *L. interrogans* Lai strain 56601**

**A:** PAS-GGDEF genes architecture is presented in an arrow diagram based on bioinformatics results. B: Multiple sequence alignment of PAS-GGDEF domain proteins. Black indicates the identical sequences, gray indicates less conserved sequences.

**Figure S2. Multiple DGCs and PDEs from *L. interrogans* affect c-di-GMP accumulation in heterologous hosts**

Visible fluorescence of DGCs and PDEs report strains were recorded by camera under a bright field. Report strains culture, induction and photography were carried out as described in material section.

**Figure S3. Heterologous expression of LA2528 and LA1483 in *B. Subtilis* affect c-di-GMP accumulation**

Engineered *B. Subtilis* NPS236 which is devoid of c-di-GMP metabolic enzymes was reported to be applied to characterization of heterologous DGCs and PDEs through observing motility changes. LA2528, LA1483 and LB237 were heterologously expressed in NPS236. A represents the image of empty NPS236. B, C and D represent the image of heterologous expression of LA1483, LB237 and LA2528 in NPS236 respectively. All the strains were cultivated in 0.5% agar LB at 37℃ overnight according to materials and methods.

**Figure S4. Determination of LA2383 PDE activity by colorimetric assays**

The purified protein mixed with bis-*p*NPP, which used as substrate in reaction buffer in incubated at 37 ℃ for two hours, then detected at 410 nm by spectrophotometer. BSA used as negative control. The error bar represents standard deviation from three independent experiments; ** indicates significant difference from the BSA (P < 0.01 by Student’s t test).

**Figure S5. Determination of the expression of DGCs and PDEs in *E. coli***

Fractions of lysates from *E. coli* BL21(DE3) cells containing the empty vector pET28a (Control) or pET28a-targetgenes were electrophoresed in a 10% SDS-PAGE gel. M indicates the protein ladder. + IPTG means samples were induced with addition of 1.0 mM IPTG. White arrows represent interest proteins.
